# Evaluation of an Automated Text Message–Based Program to Reduce Use of Acute Health Care Resources After Hospital Discharge

**DOI:** 10.1001/jamanetworkopen.2022.38293

**Published:** 2022-10-26

**Authors:** Eric Bressman, Judith A. Long, Katherine Honig, Jarcy Zee, Nancy McGlaughlin, Carlondra Jointer, David A. Asch, Robert E. Burke, Anna U. Morgan

**Affiliations:** 1 Division of General Internal Medicine, Department of Medicine, Perelman School of Medicine, University of Pennsylvania, Philadelphia; 2 Leonard Davis Institute of Health Economics, University of Pennsylvania, Philadelphia; 3 Corporal Michael J. Crescenz Veterans Affairs Medical Center, Philadelphia, Pennsylvania; 4 Department of Biostatistics, Epidemiology, and Informatics, Perelman School of Medicine, University of Pennsylvania, Philadelphia; 5 Department of Biostatistics, Epidemiology, and Informatics, Children’s Hospital of Philadelphia, Philadelphia, Pennsylvania; 6 Primary Care Service Line, University of Pennsylvania Health System, Philadelphia; 7 Center for Health Care Innovation, University of Pennsylvania Health System, Philadelphia

## Abstract

**Importance:**

Posthospital contact with a primary care team is an established pillar of safe transitions. The prevailing model of telephone outreach is usually limited in scope and operationally burdensome.

**Objective:**

To determine whether a 30-day automated texting program to support primary care patients after hospital discharge is associated with reductions in the use of acute care resources.

**Design, Setting, and Participants:**

This cohort study used a difference-in-differences approach at 2 academic primary care practices in Philadelphia from January 27 through August 27, 2021. Established patients of the study practices who were 18 years or older, were discharged from an acute care hospitalization, and received the usual transitional care management telephone call were eligible for the study. At the intervention practice, 604 discharges were eligible and 430 (374 patients, of whom 46 had >1 discharge) were enrolled in the intervention. At the control practice, 953 patients met eligibility criteria. The study period, including before and after the intervention, ran from August 27, 2020, through August 27, 2021.

**Exposure:**

Patients received automated check-in text messages from their primary care practice on a tapering schedule during the 30 days after discharge. Any needs identified by the automated messaging platform were escalated to practice staff for follow-up via an electronic medical record inbox.

**Main Outcomes and Measures:**

The primary study outcome was any emergency department (ED) visit or readmission within 30 days of discharge. Secondary outcomes included any ED visit or any readmission within 30 days, analyzed separately, and 30- and 60-day mortality. Analyses were based on intention to treat.

**Results:**

A total of 1885 patients (mean [SD] age, 63.2 [17.3] years; 1101 women [58.4%]) representing 2617 discharges (447 before and 604 after the intervention at the intervention practice; 613 before and 953 after the intervention at the control practice) were included in the analysis. The adjusted odds ratio (aOR) for any use of acute care resources after implementation of the intervention was 0.59 (95% CI, 0.38-0.92). The aOR for an ED visit was 0.77 (95% CI, 0.45-1.30) and for a readmission was 0.45 (95% CI, 0.23-0.86). The aORs for death within 30 and 60 days of discharge at the intervention practice were 0.92 (95% CI, 0.23-3.61) and 0.63 (95% CI, 0.21-1.85), respectively.

**Conclusions and Relevance:**

The findings of this cohort study suggest that an automated texting program to support primary care patients after hospital discharge was associated with significant reductions in use of acute care resources. This patient-centered approach may serve as a model for improving postdischarge care.

## Key Points

**Question:**

Is a 30-day, automated text message–based transitional care management program associated with a reduction in readmissions or emergency department visits after hospital discharge?

**Findings:**

In this cohort study, an automated text message–based program was associated with a statistically significant 41% lower odds of 30-day use of acute care resources.

**Meaning:**

These findings suggest that primary care–based transitional care management programs can consider automated texting strategies to augment support for patients after hospital discharge with limited added burden to staff.

## Teaser

This cohort study assesses whether a 30-day intervention using automated texting is associated with reductions in use of acute health care resources among patients discharged from the hospital.

## Introduction

The period after discharge from an acute care hospitalization is a vulnerable time for patients^1,2,3^ as they recover strength, learn new medication regimens, and coordinate follow-up care. A variety of postdischarge care management strategies aim to bridge gaps in care and identify needs early, often with the goal of reducing subsequent use of acute care resources.^4,5^

A common approach is to use a single primary care–based, nurse-led telephone call to identify needs shortly after discharge,^6,7,8,9^ which aligns with Medicare’s billing requirements for transitional care management (TCM) services.^10^ This approach has proven effective in some settings in reducing unplanned readmissions; however, the calls are limited in scope and present a significant operational burden.^11,12^ In our experience, the calls can be time intensive, often go unanswered, and generally connect with patients only once, early in the course of their recovery.

To overcome these limitations, we sought to test a model of postdischarge care management outreach using automated text messaging with 2-way capability. This approach has the potential to overcome several limitations of call-based programs: (1) automation allows scaled-up outreach, conserving staff time for patients with identified needs; (2) 2-way texting offers a low-friction medium for patients to initiate interactions; and (3) texting allows for asynchronous communication. Prior studies using automated calls as a tool to support postdischarge care management^13,14^ have demonstrated reductions in readmissions with limited added burden to staff. Text messaging has also been associated with higher rates of engagement compared with calls.^15^

We developed a 30-day postdischarge intervention using automated texting, implemented it in a single primary care practice, and compared the intervention practice with a control practice. We tested the hypothesis that the intervention would be associated with reductions in postdischarge use of acute care resources. In addition, we evaluated patient engagement and experience.

## Methods

### Study Design

We designed and implemented a new 30-day postdischarge intervention using automated texting to supplement the standard of care. This was implemented in a single primary care practice within Penn Medicine from January 27 through August 27, 2021. We compared changes in use of acute care resources among hospital discharges of our intervention practice and a control practice during the 30 days after discharge using a cohort study design with a difference-in-differences approach. Both practices are housed in the same building 1 floor apart, are served by Penn Medicine primary care clinicians and staff, and have similar patient populations. This study was determined by the University of Pennsylvania Institutional Review Board to meet criteria for quality improvement and therefore did not require review or informed consent. We followed the Strengthening the Reporting of Observational Studies in Epidemiology (STROBE) reporting guideline.

### Participants and Procedures

Eligible patients in both the control and intervention practices were adults (18 years and older) who were discharged from an acute care hospitalization and who were eligible for and received a TCM telephone call. Exclusion criteria for the standard-of-care TCM telephone calls are (1) planned chemotherapy admissions; (2) certain scheduled surgical procedures, including spinal surgery, joint replacements, gastric bypass, transurethral resection of the prostate, gynecological procedures, and transplants; and (3) obstetrics admissions.

Patients were identified for inclusion through the usual workflow in these practices, which use daily HealthShare Exchange reports, a health information exchange for the greater Philadelphia region that provides practices with information on discharges from all regional hospitals.^16^ Patients were then screened for exclusion criteria based on data available in these reports and, where available, electronic medical record (EMR) data. Patients could be enrolled for each discharge during the study period.

Patients from the control practice received the standard-of-care TCM telephone call from their practice within 2 business days of discharge. This call is meant to identify any needs soon after discharge and consists of a set of questions related to follow-up appointments, medications, symptoms, and home care needs. Patients were also scheduled for a postdischarge office visit during the call. If they did not answer the first call, one more attempt was made; further outreach was left to the nurses’ discretion.

Patients from the intervention practice received the same TCM telephone call with the same process and were additionally told about the texting program. They had the opportunity to verbally decline enrollment at this time. Patients identified as not having a texting-capable phone or the ability to text in English were not enrolled in the program. Patients who did not answer the TCM call were by default enrolled in the texting program at the time of the unanswered call (with the opportunity to opt out via text, as below). On enrollment (day 0), patients received an introductory message describing the program and advising them how to reach out or opt out at any time. They were asked if they had an appointment with their primary care clinician or a specialist within the next 2 weeks. If they answered no, their response was escalated back to the practice via the EMR for help in coordinating an appointment as necessary.

The day after enrollment (day 1), patients began receiving check-in messages on a tapering schedule (eTable 1 in the Supplement). These messages asked whether any help was needed. “No” responses prompted no further action. “Yes” responses were followed by a message asking the patient to categorize their need (eg, “I don’t feel well” or “I need help with my medication”) (eMethods in the Supplement). Responses were escalated back to the practice for a follow-up telephone call. Escalation messages were read only during business hours and were responded to no later than 1 business day after receipt (generally the same business day).

Patients were given the opportunity to opt out at any time. Patients who did not respond to 3 consecutive messages would receive an additional inactive check-in message asking whether they wanted to continue receiving messages or if they needed additional help; if they did not respond to this, messages would continue at their regular cadence. At the conclusion of the program (day 30), patients received a closing message and a 1-item survey (the Net Promoter Score [NPS] question) gauging their satisfaction with the program.

The texting program was built and managed by Way to Health, a National Institutes of Health–funded platform designed to provide automated technology infrastructure in support of clinical care and care delivery innovation research. Escalations were routed to a regularly monitored inbox in the EMR.

### Data Source and Collection

Demographic and clinical information was collected from Penn Medicine’s EMR (Epic Systems Corporation). These data were collected for all eligible discharges in both the intervention and control practices between August 27, 2020, and August 27, 2021 (patients could be represented more than once if they had multiple discharges during this period). The intervention was launched on January 27, 2021; the preintervention and postintervention periods were defined as discharges either before or after and including this date. User engagement and satisfaction data for those enrolled in the intervention were collected from the Way to Health platform.

### Measures

#### Demographic and Clinical Characteristics

Demographic characteristics included age, sex, race and ethnicity, and insurance payer. Sex as well as race and ethnicity were self-reported and recorded in the EMR. Race and ethnicity data were considered important in assessing any baseline demographic differences in the intervention and control populations. Clinical and risk characteristics included the Charlson Comorbidity Index, the UPHS (University of Pennsylvania Health System) risk score (an Epic Systems Corporation–developed and –validated point score used to estimate a patient’s risk of adverse health events in the next year based on clinical information presented in prior literature^17,18,19^) (details regarding the calculation of this score are provided in eTable 2 in the Supplement), and length of hospital stay.

#### Clinical Outcomes

The primary outcome was a binary composite measure of any use of acute care resources (emergency department [ED] or hospitalization) at any Penn Medicine or non–Penn Medicine facility in the HealthShare Exchange database within 30 days of discharge. Secondary outcomes included 1 or more ED visits (without subsequent hospitalization) within 30 days and 1 or more readmissions within 30 days. As a safety outcome, we also examined death within 30 and 60 days of discharge.

#### Acceptability and Feasibility Outcomes

For patients enrolled in the texting program, we tracked program uptake, use, clinical escalations, and dropout. We measured satisfaction via a score ranging from 0 (unlikely) to 10 (extremely likely) on the NPS question: “How likely are you to recommend Penn Medicine’s discharge follow-up program to a friend or colleague?”^20^ The NPS is reported as an integer value from −100 to 100, with higher values indicating greater likelihood to recommend the program (further details on scoring are provided in the eMethods in the Supplement).

### Statistical Analysis

We compared changes in 30-day use of acute care resources (both the composite measure and ED visits and readmissions separately) using a difference-in-differences approach, excluding patients who died within 30 days of discharge. To avoid selection bias (ie, because there was no assessment of texting capabilities or other mitigating factors at the control practice), we used an intention-to-treat approach in the analyses: all patients to whom a TCM telephone call was placed (whether or not it was answered) were included in the analysis of the effectiveness outcomes at both the intervention and the control practices regardless of whether they were successfully enrolled in the texting program.

To visually inspect the data for preintervention parallel trends while limiting the large variation in monthly rates of 30-day use of acute care resources, we calculated a 3-month rolling mean. In addition, we conducted formal statistical testing for parallel trends by using a logistic regression model with an interaction between practice and time before the start of the intervention. We used a logistic generalized estimating equation model for each outcome (Stata package GEE, independent correlation structure). The difference-in-differences was estimated by the coefficient for the interaction term between the period (preintervention and postintervention start date) and the practice (intervention and control). We adjusted for patient demographic characteristics (age, sex, race and ethnicity, and insurance payer), risk estimators (UPHS risk score and Charlson Comorbidity Index), and length of hospital stay. We used similar models to assess differences in death outcomes within 30 and 60 days of discharge.

All statistical analyses used Stata software, version 16.1 (StataCorp LLC). Two-sided *P* < .05 indicated statistical significance.

### Sensitivity Analysis

To ensure that our findings were not due to specific trends at the preselected control practice, we extracted data for 5 additional practices in Philadelphia. Using these data, we conducted a secondary analysis with the same model but with a larger control group consisting of 6 total practices (eTable 5 in the Supplement).

## Results

The study sample included 1885 unique patients (771 from the intervention practice and 1114 from the control practice), with a total of 2617 discharges (447 before and 604 after the intervention at the intervention practice; 613 before and 953 after intervention at the control practice). Among the 1885 patients, the mean (SD) age was 63.2 (17.3) years; 1101 (58.4%) were women and 784 (41.6%) were men; 1034 (54.9%) were White; and 976 (51.8%) were insured by Medicare. The sample at the intervention practice had a higher proportion of women (501 [65.0%] vs 600 [53.9%]) and Black patients (364 [47.2%] vs 390 [35.0%]) and had slightly higher UPHS risk scores (mean [SD], 3.8 [2.4] vs 3.3 [2.1]). Demographically, patients in the intervention and control practices were otherwise broadly similar (Table 1).

**Table 1.** Patient Characteristics Across the Entire Period of Analysis

Characteristic	Patient group^a^			*P* value
	All (N = 1885)	Intervention practice (n = 771)	Control practice (n = 1114)	
Sex				
Women	1101 (58.4)	501 (65.0)	600 (53.9)	<.001
Men	784 (41.6)	270 (35.0)	514 (46.1)	
Age, mean (SD), y	63.2 (17.3)	63.5 (16.8)	62.9 (17.6)	.41
Race				
Black	754 (40.0)	364 (47.2)	390 (35.0)	<.001
White	1034 (54.9)	362 (47.0)	672 (60.3)	
Other or unknown^b^	97 (5.2)	45 (5.8)	52 (4.7)	
Ethnicity				
Hispanic	85 (4.5)	32 (4.1)	53 (4.8)	.53
Non-Hispanic	1800 (95.5)	739 (95.8)	1061 (95.2)	
Payor				
Commercial	775 (41.1)	307 (39.8)	468 (42.0)	.58
Medicare	976 (51.8)	403 (52.3)	573 (51.4)	
Medicaid	114 (6.0)	52 (6.7)	62 (5.6)	
Self-pay	20 (1.1)	9 (1.2)	11 (1.0)	
UPHS risk score, mean (SD)	3.5 (2.2)	3.8 (2.4)	3.3 (2.1)	<.001
Charlson Comorbidity Index, mean (SD)	5.4 (4.0)	5.5 (3.9)	5.3 (4.0)	.46
Length of stay, mean (SD), d	4.1 (5.7)	4.1 (4.8)	4.1 (6.2)	.97

Abbreviation: UPHS, University of Pennsylvania Health System.

^a^ Observations represent unique patients; 425 patients had more than 1 discharge during the study period, which were included and accounted for in the analysis. Unless otherwise indicated, data are expressed as No. (%) of patients. Percentages have been rounded and may not total 100.

^b^ Includes American Indian or Alaska Native, Asian, East Indian, Pacific Islander, and those who self-described as “other.”

**Figure 1.**
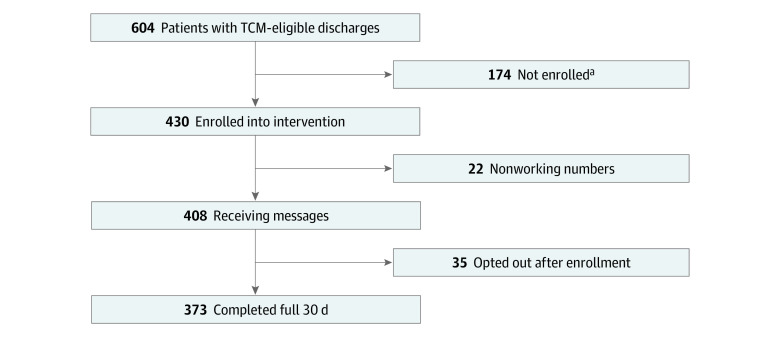
Study Flow Diagram TCM indicates transitional care management. ^a^Reasons included no texting capable phone, unable to text in English, or declined enrollment during call (patient subset numbers are unavailable).

Of the 604 eligible discharges during the intervention period, 430 were from patients enrolled in the intervention (374 unique patients, of whom 46 had multiple enrollments) (Figure 1). Of the total enrollments, 22 (5.1%) had a nonworking telephone number. Of the 408 enrollments with working numbers, 35 (8.6%) subsequently opted out before the end of the 30 days.

### Clinical Outcomes

**Figure 2.**
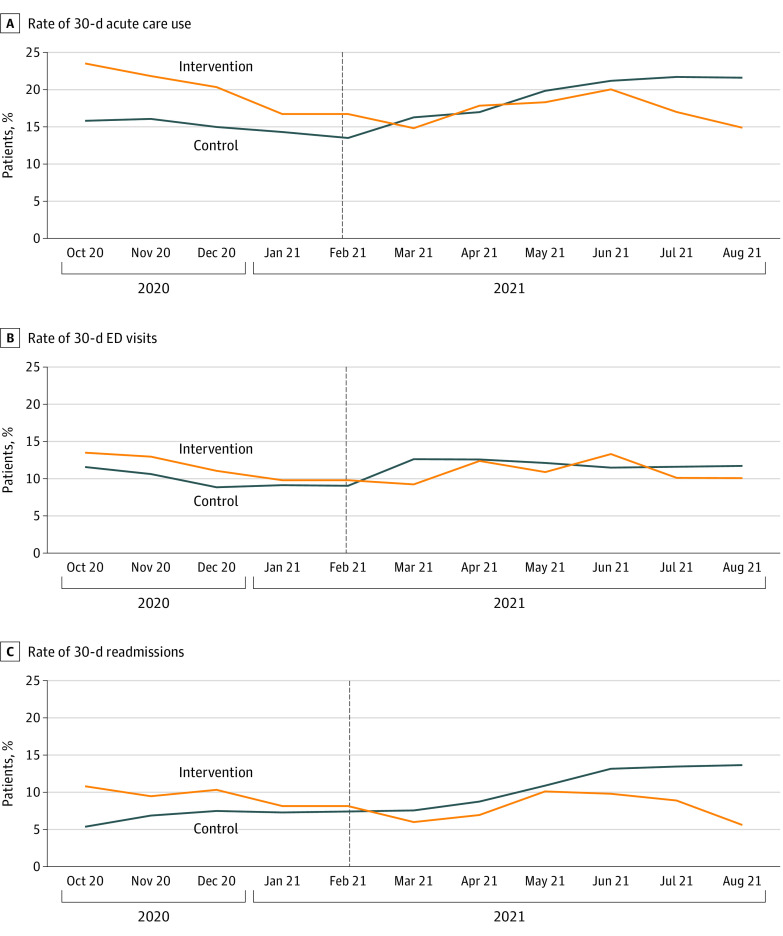
Trends in Rate of 30-Day Postdischarge Use of Acute Care Resources, Presented as a Rolling 3-Month Mean by Month The black vertical line indicates the start of the pilot program. ED indicates emergency department.

In the months before the intervention, the trends in 30-day use of acute care resources were similar between the 2 practices (Figure 2 and the eFigure in the Supplement). A regression model for each outcome in the preintervention period revealed no interaction between practice and time (eTable 3 in the Supplement).

The 30-day rate of use of acute care resources changed from 15.2% before the intervention to 19.8% after the intervention for the control practice and from 20.3% to 16.5%, respectively, for the intervention practice. In the adjusted model, the odds of 30-day use of acute care resources after implementation of the intervention were 41% lower (adjusted odds ratio [aOR], 0.59 [95% CI, 0.38-0.92]; *P* = .02) at the intervention practice compared with the control practice (Table 2). For an ED visit within 30 days, the odds were 33% lower; however, this result was not significant (aOR, 0.77 [95% CI, 0.45-1.30]; *P* = .33). For a readmission within 30 days, the odds were 55% lower (aOR, 0.45 [95% CI, 0.23-0.86]; *P* = .01).

**Table 2.** 30-Day Rates of Use of Acute Care Resources by Practice Before and After the Intervention^a^

Outcome	No. of discharges/total No. (%)		Difference-in-differences, aOR (95% CI)
	Before intervention	After intervention	
Use of acute care resources			
Control practice	93/606 (15.3)	183/945 (19.4)	0.59 (0.38-0.92)
Intervention practice	86/435 (19.8)	98/595 (16.5)	
ED visit			
Control practice	62/606 (10.2)	110/945 (11.6)	0.77 (0.45-1.30)
Intervention practice	49/435 (11.3)	63/595 (10.6)	
Readmission			
Control practice	42/606 (6.9)	107/945 (11.3)	0.45 (0.23-0.86)
Intervention practice	42/435 (9.7)	44/595 (7.4)	

Abbreviations: aOR, adjusted odds ratio; ED, emergency department.

^a^ The before-intervention period includes August 27, 2020, to January 26, 2021; the after-intervention period, January 27 to August 27, 2021.

The aORs for death within 30 and 60 days of discharge at the intervention practice were 0.92 (95% CI, 0.23-3.61; *P* = .91) and 0.63 (95% CI, 0.21-1.85; *P* = .40), respectively (eTable 4 in the Supplement). The sensitivity analysis including 5 additional control practices revealed an aOR for use of acute care resources of 0.67 (95% CI, 0.47-0.95); for an ED visit, 0.67 (95% CI, 0.43-1.04); and for a readmission, 0.70 (95% CI, 0.44-1.12) (eTable 5 in the Supplement).

### Engagement and Satisfaction

There were 301 escalations during the course of the intervention (mean [SD], 0.70 [1.04] escalations per enrollment) for a mean (SD) of 1.4 (1.5) per day. Of these, 260 (86.4%) were in response to an automated check-in and 41 (13.6%) were initiated by the patient outside a regular check-in window. A total of 338 participants (82.8%) responded to either the introductory message or the first check-in message. Of those who received messages for the full 30 days (n = 373), 165 (44.2%) responded to the closing NPS question. The NPS was +67.

## Discussion

In this cohort study, a text message–based automated system of monitoring patients after hospitalization was associated with a 41% reduction in the odds of 30-day use of acute care resources, suggesting that a 30-day automated texting program can improve postdischarge outcomes among primary care patients. This outcome was driven largely by a 55% decrease in the odds of a 30-day readmission. The program’s high degree of automation required minimal effort beyond usual care. To the best of our knowledge, this is the first study to experimentally test the benefit of an automated texting program on postdischarge outcomes among primary care patients.^21^ The mechanism through which this compound program prevents use of acute care is likely complex, but we theorize that more frequent check-ins and a lower friction medium for patient-initiated outreach lead to earlier identification of needs and a greater likelihood that issues will be escalated to and handled by the primary care practice than another setting.^22^

This study builds on a growing body of literature around the utility of automated hovering and text message–based strategies to support patients in the transition from hospital to home.^23,24,25,26,27,28^ Prior studies have evaluated these approaches in a variety of clinical contexts, including after surgery and in specific disease states, such as heart failure. Of those studies that have evaluated the association with the use of acute care resources, the findings have been mixed, with some finding no significant difference,^23,24^ and others similarly finding an associated reduction in readmissions.^25^ Our study is, to our knowledge, the first to evaluate a text message–based, automated hovering approach in a broad primary care population.

In addition to the associated reduction in the use of acute care resources, this program was highly acceptable to patients: 82.8% of patients responded to at least 1 of our introductory messages—far higher than rates of response to our traditional transitions calls. This outcome is consistent with other work that has compared text messaging with traditional call-based approaches.^29^ Only 8.6% of those enrolled opted out of our program. The NPS of those who remained was +67, which is considered excellent.^30^ High rates of satisfaction has been a consistent finding in other similar programs.^26,27^

Last, our intervention was integrated into the preexisting workflow of the practice care management team, for whom responding to patient calls from recently discharged patients is part of their daily standard work. The intervention was integrated into these existing structures and generated a mean of 1.4 escalations per day, which the team considered a manageable workload. Although the staff continued to place the standard telephone calls during this pilot, future work can test replacement of these calls with messaging entirely.

### Limitations

This study has some limitations. It was deployed at a single site, and patients were not randomized. Patients may have been excluded from the intervention based on considerations not readily identifiable in the EMR. We accounted for this factor by including all patients who received a TCM telephone call in our analysis (intention-to-treat approach), which should eliminate potential selection bias between the intervention and control groups and, if anything, bias our results toward the null. We included mortality as a secondary, safety outcome, although we were limited to information available in our EMR, which is not always complete. This study occurred during the COVID-19 pandemic, which may have enhanced uptake of the intervention, as it was a time when practices and patients became more accustomed to remote outreach and virtual care. This factor is not likely to limit generalizability going forward, because these features have now become more standard in the primary care toolbox. Net promoter scores may have been influenced by selection of those who completed the 30 days of the study and chose to respond. Texting, although generally widespread and accessible,^31^ may pose barriers for some patients, particularly in terms of cost for those who do not have unlimited texting plans. However, past work^32^ has indicated that technology can also reduce disparities, especially for postdischarge care. Additionally, despite adjustment for several covariates, this nonrandomized assessment is at risk of residual confounding. These findings are encouraging and warrant confirmation in a randomized study.

## Conclusions

The findings of this cohort study suggest that an automated texting program to support care management for primary care patients after hospital discharge is highly feasible and acceptable. The program was associated with significant reductions in the use of acute care resources during the 30 days after discharge.

## Notes

Supplement. **eTable 1.** Schedule of Outreach **eMethods.** Script of Messages and Net Promoter Score **eTable 2.** Calculation of UPHS Risk Score **eFigure.** Trends in 30-Day Acute Care Resource Use Presented as Monthly Average **eTable 3.** Parallel Trends Testing Before Intervention **eTable 4.** Mortality Outcomes **eTable 5.** Sensitivity Analysis
