# Supplementary material for: Evaluation of an Automated Text Message–Based Program to Reduce Use of Acute Health Care Resources After Hospital Discharge
Source: JAMA Netw Open. 2022 Oct 26;5(10):e2238293. doi: 10.1001/jamanetworkopen.2022.38293 (PMC9606844; doi:10.1001/jamanetworkopen.2022.38293)
Supplement: Supplement. — eTable 1. Schedule of Outreach eMethods. Script of Messages and Net Promoter Score eTable 2. Calculation of UPHS Risk Score eFigure. Trends in 30-Day Acute Care Resource Use Presented as Monthly Average eTable 3. Parallel Trends Testing Before Intervention eTable 4. Mortality Outcomes eTable 5. Sensitivity Analysis [file jamanetwopen-e2238293-s001.pdf]

Supplementary Online Content

Bressman E, Long JA, Honig K, et al. Evaluation of an automated text message–based program to reduce use of acute health care resources after hospital discharge. *JAMA Netw Open*. 2022;5(10):e2238293. doi:10.1001/jamanetworkopen.2022.38293

eTable 1. Schedule of Outreach
eMethods. Script of Messages and Net Promoter Score
eTable 2. Calculation of UPHS Risk Score
eFigure. Trends in 30-Day Acute Care Resource Use Presented as Monthly Average
eTable 3. Parallel Trends Testing Before Intervention
eTable 4. Mortality Outcomes
eTable 5. Sensitivity Analysis

This supplementary material has been provided by the authors to give readers additional information about their work.

© 2022 Bressman E et al. *JAMA Network Open*.

eTable 1. Schedule of Outreach

Practice	Within 2 business days of discharge	Week 1	Week 2	Week 3	Week 4	Day 30
Control	Standard TCM phone call from the practice	X	X	X	X	X
Intervention	Standard TCM phone call from the practice<br>Enrollment in automated texting platform<br>Appointment question	Three check-in messages	Two check-in messages	One check-in message	One check-in message	Closing message

eMethods. Script of Messages and Net Promoter Score

- Enrollment messages
    - Hello {name}. The care manager from {PCP's name}'s office enrolled you in this Penn Medicine texting program. This program is for people who have been recently discharged from the hospital.
    - We will check in regularly to see how you are doing and make sure you get the care you need. We will respond to you within 1 business day. Please note that texting is not 100% secure. Message & data rates may apply.
    - If you want to speak with us in between check-ins, text "CALL". If you need immediate medical help, call 911. If you do not want to receive these messages, text "BYE" at any time.
- Appointment question
    - Do you have an appointment with you Primary Care doctor or specialist within the next 1-2 weeks?
- Regular check-in question
    - Is there anything we can help you with today?
        - If patient answers "yes": Thanks for reaching out. Before we call we'd like to get a little more information. What can we help you with?
            - A) I don't feel well
            - B) I need help with my medicines
            - C) I need help with my appointments
            - D) I need help at home
            - E) More than one or something else
- Closing message
    - You have reached the end of the 30-day discharge follow-up program. You will no longer receive messages from us. If you have a new need or a non-urgent medical issue, call your doctor. If you need immediate medical help, call 911.
- Net Promoter Score question

© 2022 Bressman E et al. *JAMA Network Open*.

- On a scale of 0 (unlikely) to 10 (extremely likely), how likely are you to recommend Penn Medicine's discharge follow up program to a friend or colleague?

Calculation of Net Promoter Score (NPS)

- NPS = % promoters - % detractors
    - "Promoters" are those who give a score of 9-10
    - "Detractors" are those who give a score of 0-6
    - "Passives" are those who give a score of 7-8
- The score is reported as an integer value from -100 to +100

eTable 2. Calculation of UPHS Risk Score

**Risk Factor**	**1 Point**	**2 Points**	**3 Points**
Age	65-84	85+	
Number of acute admissions in the past year	1 admission	2 admissions	3+ admissions
Number of ED visits in the past year	1 visit	2 visits	3+ visits
COPD, CHF, Diabetes, or Chronic Liver Disease	1 point per diagnosis		
Depression	Has diagnosis of depression		
No PCP	Patient doesn't have a PCP		
Medicaid	Patient is on Medicaid		

*Scored from 0-15

© 2022 Bressman E et al. *JAMA Network Open*.

eFigure. Trends in 30-Day Acute Care Resource Use Presented as Monthly Average

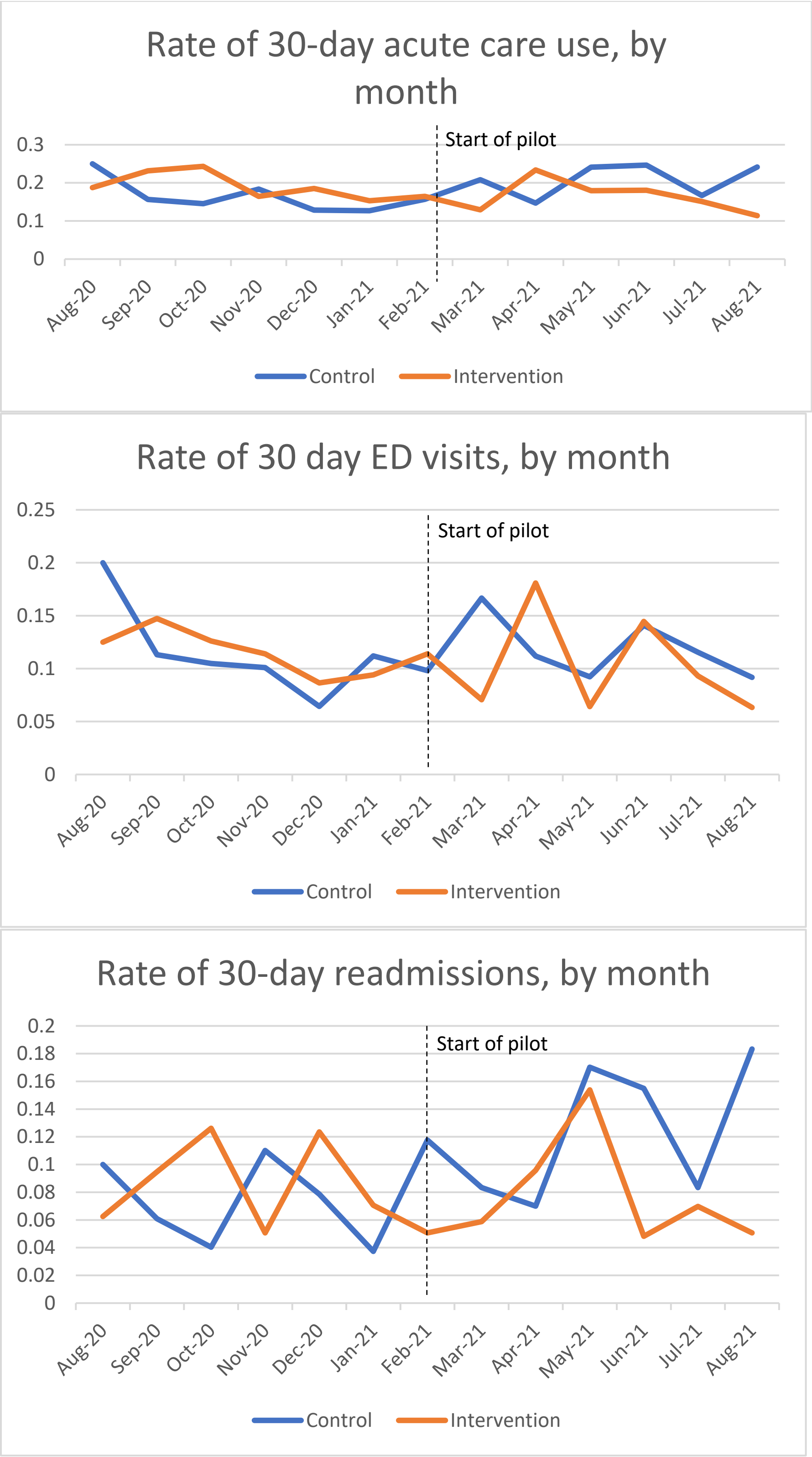

© 2022 Bressman E et al. *JAMA Network Open*.

eTable 3. Parallel Trends Testing Before Intervention

**Outcome**	**Odds Ratio Estimate**	**95% CI**	**p-value**
Acute care utilization	0.95	0.76 - 1.20	0.70
ED visit	0.89	0.68 - 1.18	0.43
Readmission	1.20	0.87 - 1.68	0.29
Death within 30 days	0.93	0.47 - 1.83	0.83
Death within 60 days	0.75	0.42 - 1.36	0.34

eTable 4. Mortality Outcomes

Outcome		Before period (%)	After period (%)	Difference-in-difference (aOR, 95% CI)
30-day mortality	Control	7/613 (1.1%)	8/953 (0.8%)	0.92 (0.23 - 3.61)
	Intervention	12/447 (2.7%)	9/604 (1.5%)	
60-day mortality	Control	10/613 (1.6%)	17/953 (1.8%)	0.63 (0.21 - 1.85)
	Intervention	18/447 (4.0%)	15/604 (2.5%)	

eTable 5. Sensitivity Analysis

Primary regression model incorporating additional control practices, for a total of 6 control practices

Outcome		Before period (%)	After period (%)	Difference-in-difference (aOR, 95% CI)
Acute care utilization	Control	695/3331 (20.9%)	1253/5504 (22.77%)	0.67 (0.47 – 0.95)
	Intervention	86/435 (19.8%)	98/595 (16.5%)	
ED visit	Control	364/3331 (10.9%)	724/5504 (13.2%)	0.67 (0.43 – 1.04)
	Intervention	49/435 (11.3%)	63/595 (10.6%)	
Readmission	Control	414/3331 (12.4%)	711/5504 (12.9%)	0.70 (0.44 – 1.12)
	Intervention	42/435 (9.7%)	44/595 (7.4%)	

© 2022 Bressman E et al. *JAMA Network Open*.
